# P2X1 Receptors Amplify FcγRIIa-Induced Ca ^2+^ Increases and Functional Responses in Human Platelets

**DOI:** 10.1160/TH17-07-0530

**Published:** 2018-01-29

**Authors:** Zeki Ilkan, Stephanie Watson, Steve P. Watson, Martyn P. Mahaut-Smith

**Affiliations:** 1 Department of Molecular and Cell Biology, Henry Wellcome Building, University of Leicester, Leicester, United Kingdom; 2 Institute of Cardiovascular Sciences, Institute of Biomedical Research Building, University of Birmingham, Birmingham, United Kingdom; 3 Centre of Membrane Proteins and Receptors (COMPARE), Universities of Birmingham and Nottingham, Midlands, UK

**Keywords:** bacteria, immunity, inflammation, thrombosis, platelet

## Abstract

Platelets express key receptors of the innate immune system such as FcγRIIa and Toll-like receptors (TLR). P2X1 cation channels amplify the platelet responses to several major platelet stimuli, particularly glycoprotein (GP)VI and TLR2/1, whereas their contribution to Src tyrosine kinase-dependent FcγRIIa receptors remains unknown. We investigated the role of P2X1 receptors during activation of FcγRIIa in human platelets, following stimulation by cross-linking of an anti-FcγRIIa monoclonal antibody (mAb) IV.3, or bacterial stimulation with *Streptococcus sanguinis* . Activation was assessed in washed platelet suspensions via measurement of intracellular Ca ^2+^ ([Ca ^2+^ ] _i_ ) increases, ATP release and aggregation. P2X1 activity was abolished by pre-addition of α,β-meATP, exclusion of apyrase or the antagonist NF449. FcγRIIa activation evoked a robust increase in [Ca ^2+^ ] _i_ (441 ± 33 nM at 30 μg/mL mAb), which was reduced to a similar extent (to 66–70% of control) by NF449, pre-exposure to α,β-meATP or apyrase omission, demonstrating a significant P2X1 receptor contribution. FcγRIIa activation-dependent P2X1 responses were partially resistant to nitric oxide (NO), but abrogated by 500 nM prostacyclin (PGI _2_ ). Aggregation responses to bacteria and FcγRIIa activation were also inhibited by P2X1 receptor desensitization (to 66 and 42% of control, respectively). However, FcγRIIa-mediated tyrosine phosphorylation and ATP release were not significantly altered by the loss of P2X1 activity. In conclusion, we show that P2X1 receptors enhance platelet FcγRIIa receptor-evoked aggregation through an increase in [Ca ^2+^ ] _i_ downstream of the initial tyrosine phosphorylation events and early dense granule release. This represents a further route whereby ATP-gated cation channels can contribute to platelet-dependent immune responses in vivo.

## Introduction

In addition to their essential role in the process of haemostasis, platelets contribute to immune responses through several mechanisms including the interaction of surface receptors with invading pathogens. Human platelets express the low affinity receptor for immunoglobulin G, FcγRIIa (CD32a), which recognizes the immunoglobulin G (IgG) that opsonizes invading pathogens in the circulation. 1 Cross-linking of FcγRIIa receptors results in the activation of a signal transduction pathway through an immunoreceptor tyrosine-based activation motif (ITAM) in a manner similar to that observed following stimulation of the collagen and fibrin receptor GPVI. 2 The vital role of FcγRIIa receptor in platelet aggregation and thrombus formation has been established by several in vitro and in vivo studies. 3 4 5 In addition, interaction between bacteria and platelets has been shown to cause formation of dangerous circulating or localized thrombi such as in infective endocarditis (IE). 6 Despite this, our knowledge of FcγRIIa receptor involvement in platelet function remains rudimentary.

P2X1 channels are the only adenosine triphosphate (ATP)-activated receptors in platelets and represent the fastest Ca ^2+^ entry route following ATP release from an injury site. 7 The contribution of P2X1 channels to thrombosis in vivo, and their important role in primary and secondary agonist-induced platelet activation, has been described previously. 8 9 It has been shown that selective inhibition or desensitization of P2X1 channels reduces the [Ca ^2+^ ] _i_ increases triggered by Toll-like receptors 2/1 (TLR2/1) 10 with the synthetic triacylated lipopeptide Pam _3_ CSK _4_ , and several natural platelet agonists such as thrombin, thromboxane A _2_ , adenosine 5′-diphosphate (ADP) and collagen. 8 This amplification of Ca ^2+^ entry likely explains the ability of P2X1 receptors to amplify functional responses, particularly at low levels of stimulation. 11 12 Importantly, P2X1 activity linked to the activation of both TLRs and GPVI was found to persist when endothelium-derived inhibitory molecules such as NO and prostacyclin (PGI _2_ ) were present in the extracellular milieu, highlighting the unique contribution of this ligand-gated cation channel to thrombosis and its potential as a drug target. 10 The ability of P2X1 receptors to contribute so efficiently to platelet responses likely results from their rapid activation mechanism and predominantly autocrine stimulation by ATP released from dense granules. 8 However, it is unknown whether this contribution of P2X1 receptors to GPVI and TLR2/1 responses modifies the early tyrosine kinase-dependent steps. Furthermore, the relative importance of P2X1 channels to FcγRIIa receptor platelet signalling and downstream responses is unknown.

In the present study, we provide evidence that human platelet P2X1 receptors contribute to the [Ca ^2+^ ] _i_ increase and aggregation following FcγRIIa receptor activation achieved by receptor cross-linking using selective antibodies or *Streptococcus sanguinis* 133–79. 5 13 This amplification of FcγRIIa receptor-evoked Ca ^2+^ increases by P2X1 persists in the presence of high levels of the ectonucleotidase apyrase and nitric oxide (NO) and thus provides a mechanism whereby Ca ^2+^ entry may be stimulated by antibody complexes or opsonized bacteria in the intact circulation.

## Materials and Methods

### Reagents

Anti-FcγRIIa monoclonal antibody (mAb) IV.3 was purified in the laboratory from a hybridoma. Goat anti-mouse IgG F(ab′) _2_ was purchased from Fisher Scientific (UK). Apyrase (grade VII) from potato, a form of ecto-nucleoside triphosphate diphosphohydrolase (NTPDase), which displays similar properties to human CD39, 14 was from Sigma-Aldrich (Poole, UK). Spermine NONOate was from Enzo Life Sciences Ltd (Exeter, UK). The GPIIb/IIIa inhibitor eptifibatide was from Source Bioscience (Nottingham, UK). Extracellular ATP measurements were performed using firefly luciferin-luciferase (Chrono-Lume reagent kit #395; Chrono-Log Corporation, Havertown, Pennsylvania, United States). Rabbit anti-human phospho-Syk (Tyr 525/526) mAb and rabbit anti-human phospho-PLCγ2 (Tyr 1217) polyclonal antibody (pAb) were from Cell Signaling Technology (Danvers, Massachusetts, United States). Rabbit anti-human phospho-LAT (Tyr 200) mAb was from Abcam (Cambridge, UK). Mouse anti-human anti-phosphotyrosine (clone 4G10) mAb was from Millipore UK Ltd (Watford, UK). Rabbit anti-human PLCγ2 antibody (Q-20) was from Santa Cruz Biotechnology (Heidelberg, Germany). Unless otherwise stated, all reagents were from Sigma-Aldrich.

### Bacterial Culture and Preparation

*Streptococcus sanguinis* 133–79 was provided by Prof Mark Herzberg (University of Minnesota). Bacteria were cultured under anaerobic conditions at 37°C overnight and suspensions were prepared as previously described. 15 After washing in phosphate-buffered saline (PBS), the optical density of the suspension was adjusted to 1.6 at a wavelength of 600 nm, which corresponds to 6 × 10 ^8^ CFU/mL as previously shown. 5

### Preparation of Washed Platelets and FcγRIIa Receptor Stimulation

Blood was collected by venepuncture in accordance with the Declaration of Helsinki from informed consenting volunteers. The study was approved by the University of Leicester College of Life Sciences Committee for Research Ethics concerning human subjects (non-NHS). Acid-citrate-dextrose (ACD; in mM: 85 trisodium citrate, 78 citric acid, 111 glucose) was used as the anticoagulant at a ratio of 6:1 (blood:ACD). After centrifugation at 700 *g* for 5 minutes, platelet-rich plasma was removed and treated with apyrase (grade VII; 0.32 U/mL) to prevent P2X1 receptor desensitization, except where stated. Aspirin (100 μM) was also added to inhibit cyclooxygenase for studies of mAb IV.3-induced Ca ^2+^ increases to allow comparison with previous studies, particularly the relative contribution of P2X1 receptors and effects of endogenous antiplatelet reagents, 8 but was omitted from all other experiments. Platelets were loaded with Fura-2 by incubation with 2μM Fura-2AM for 45 minutes at 37°C, then centrifuged for 20 minutes at 350 *g* followed by resuspension in nominally Ca ^2+^ -free saline (in mM: 145 NaCl, 5 KCl, 1 MgCl _2_ , 10 HEPES, 10 glucose, pH 7.35 with NaOH), which also contained apyrase (0.32U/mL) except where stated. Platelet responses were measured at the same density as in whole blood. Thirty seconds prior to receptor stimulation, 2 mM CaCl _2_ was added to each cuvette. FcγRIIa receptors were stimulated either by cross-linking or bacteria. For cross-linking, platelets were pre-incubated with mAb IV.3 (1 μg/mL) for 2 minutes before addition of IgG F(ab′) _2_ . For stimulation by bacteria, platelets were incubated with pooled human IgG (0.1 mg/mL) in the cuvette prior to addition of a 10-fold dilution of the 6 × 10 ^8^ CFU/mL (see above) bacteria stock.

### Ratiometric [Ca ^2+^ ] _i_ Measurements

Ratiometric [Ca ^2+^ ] _i_ measurements were performed in a Cairn spectrophotometer system (Cairn Research Limited, Faversham, Kent, UK) at 37°C and expressed as [Ca ^2+^ ] _i_ using a dissociation constant for Ca ^2+^ of 224nM, as previously described. 8

### Aggregometry and Luminescence Measurement of ATP Secretion

Aggregation of washed platelet suspensions was assessed by standard light transmission measurements, with simultaneous luminescence measurements of ATP secretion as required, in a Chrono-Log 400 Lumi-Aggregometer (Chrono-Log Corporation), at 37°C as described in detail elsewhere. 8 14 100 μg/mL fibrinogen and 2 mM CaCl _2_ were added to the cuvette at the start of each experiment. For each batch of luciferin-luciferase, a concentration–response curve across a range of known ATP concentrations (nM) was constructed using the background-corrected peak signal (mV) detected in normal platelet saline containing 0.32 U/mL apyrase (to mimic the conditions used to assess platelet-dependent ATP release).

### Protein Phosphorylation

Protein lysates were prepared from 500 μL samples of stirred platelet suspensions, which were pretreated with 9 μM eptifibatide to prevent aggregation. Lysis was achieved by addition of an equal volume of ice-cold 2 × radioimmunoprecipitation assay (RIPA) buffer (including 1× Roche protease inhibitor tablet, 4 mM sodium orthovanadate and 20 mM sodium fluoride) 60 seconds after FcγRIIa cross-linking using IgG F(ab′) _2_ (15 μg/mL). Protein quantification was performed with a Bradford assay. Tyrosine phosphorylation was detected by Western blotting with anti-phosphotyrosine (clone 4G10; 1:1,000), phospho-Syk (Tyr 525/526; 1:500), phospho-LAT (Tyr 200; 1:1,000) and phospho-PLCγ2 (Tyr 1217; 1:250) antibodies. Membranes were re-probed with a pan-anti-PLCγ2 antibody (1:200). Lysate (20 μg) was added in each well of a 12-well Bolt 4 to 12% Bis-Tris Plus Gel (Invitrogen, Paisley, UK). Each gel included at least one lane of 10 μL of Color Prestained Protein Standard (11–245 kDa; NEB, Ipswich, Massachusetts, United States). Gels were run at 100V for 90 minutes and semi-dry transfers onto PVDF membranes were performed using a Trans-Blot Turbo Transfer Imaging System(Bio-Rad, Hertfordshire, UK). Bands were visualized using horseradish peroxidase (HRP)-conjugated secondary antibodies and an ECL Prime kit (GE Healthcare, Buckinghamshire, UK) according to the manufacturer's instructions. The membranes were placed in a hyperfilm cassette, and hyperfilms (GE Healthcare) were exposed to the membranes in a dark room. Films were developed in a hyperfilm processor and scanned for analysis.

### Statistical Analysis

All traces are representative of experiments from at least three separate donors. Average results have been expressed as means ± standard error of the mean (SEM). All statistical analyses were performed on GraphPad Prism 6.0 software (La Jolla, California, United States). Where appropriate, one-way ANOVA followed by Holm-Sidak's post hoc multiple comparisons, two-way ANOVA followed by Bonferroni's multiple comparisons tests or two-tailed paired Student's *t* -tests were used. *p* -Values of * *p* < 0.05, ** *p* < 0.01, *** *p* < 0.001 and **** *p* < 0.0001 were considered statistically significant.

## Results

### P2X1 Receptors Contribute to FcγRIIa Receptor-Evoked Ca ^2+^ Responses

**Fig. 1:**
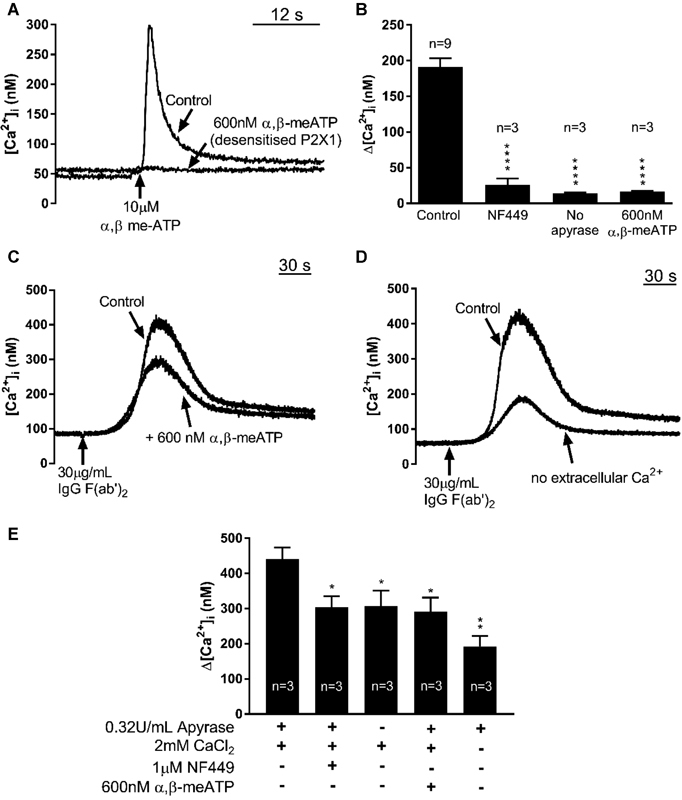
Inhibition or desensitization of P2X1 channels reduces [Ca ^2+^ ] _i_ responses induced by FcγRIIa receptor activation. ( **A** ) Representative Ca ^2+^ responses to P2X1 receptor stimulation with a supramaximal concentration (10 μM) of α,β-meATP, with or without desensitization of P2X1 channels by the pre-addition of α,β-meATP (600 nM) 90 seconds prior to stimulation. ( **B** ) Average maximal Ca ^2+^ responses to 10 μM α,β-meATP, with and without P2X1 channel inhibition by 1 μM NF449, exclusion of extracellular apyrase, or pre-exposure to 600 nM α,β-meATP. Representative ( **C, D** ) and average ( **E** ) Ca ^2+^ responses to FcγRIIa receptor activation via cross-linking of the receptor-bound mAb IV.3 (1 μg/mL), which was added 2 minutes prior to the cross-linker IgG F(ab′) _2_ antibody. The effects of the presence of NF449 (1 μM), exclusion of extracellular apyrase and Ca ^2+^ , and addition of α,β-meATP (600 nM) 90 seconds prior to IgG F(ab′) _2_ were studied.

To characterize [Ca ^2+^ ] _i_ increases induced by FcγRIIa receptor stimulation, washed platelets were pre-incubated with mAb IV.3 which permitted cross-linking of FcγRIIa 16 upon addition of IgG F(ab′) _2._ This resulted in an increase in [Ca ^2+^ ] _i_ in all platelet samples tested with a peak value of 440 ± 33 nM. Three different approaches that caused abrogation of P2X1 channel activity 8 (1 μM NF449, omission of apyrase from the platelet saline and 600 nM α,β-meATP added prior to 2 mM external Ca ^2+^ ; Fig. 1 A , B ) caused a significant and similar reduction in the peak FcγRIIa-induced [Ca ^2+^ ] _i_ increase ( Fig. 1 C , E ). The average peak FcγRIIa receptor-evoked responses were reduced to 303 ± 31 nM (69% of control; *p* < 0.05, *n* = 3) by NF449; 307 ± 42 nM (70% of control; *p* < 0.05, *n* = 3) without apyrase; and 291 ± 40 nM (66% of control; *p* < 0.05, *n* = 3) after pre-addition of 600 nM α,β-meATP (by one-way ANOVA, followed by Holm-Sidak's post hoc multiple comparisons). A further reduction of FcγRIIa receptor-evoked responses (to 191 ± 30 nM; 44% of control; *p* < 0.05, *n* = 3, by one-way ANOVA, followed by Holm-Sidak's post hoc multiple comparisons) was observed in nominally Ca ^2+^ -free saline, indicating that other Ca ^2+^ entry pathways such as store-operated Orai1 channels or TRPC6 also contribute ( Fig. 1 D , E ). As used previously for GPVI and TLR2/1 receptors, 8 10 subsequent experiments used pre-addition of 600 nM α,β-meATP to selectively abrogate P2X1 activity, as it is slightly more effective than 1 μM NF449 and small effects on P2Y receptors have been reported for higher NF449 concentrations or omission of apyrase. 8 17

**Fig. 2:**
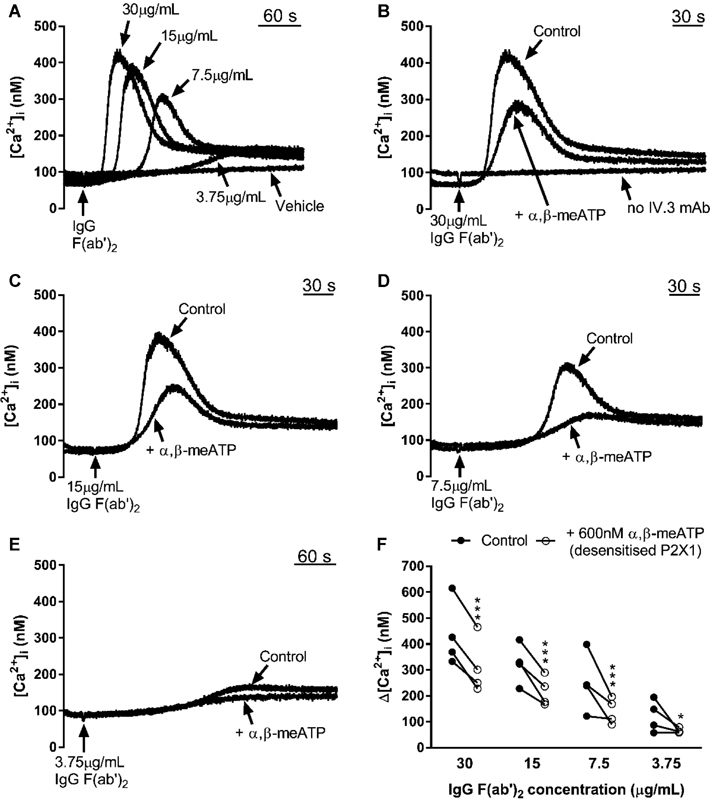
P2X1 receptor desensitization inhibits Ca ^2+^ entry through P2X1 channels induced by a range of cross-linker IgG F(ab′) _2_ antibody concentrations. Representative Ca ^2+^ responses ( **A–E** ) and individual Δ[Ca ^2+^ ]i values ( **F** ) measured in Fura-2-loaded platelet suspensions. ( **A** ) Ca ^2+^ responses obtained when a range of IgG F(ab′) _2_ concentrations were added to platelet suspensions which contained mAb IV.3 (1 μg/mL). ( **B–E** ) The effect of pretreatment of platelet suspensions with 600 nM α,β-meATP for 90 seconds on Ca ^2+^ entry following addition of different IgG F(ab′) _2_ concentrations. ( **F** ) Comparison of peak [Ca ^2+^ ] _i_ increases following cross-linking of mAb IV.3 with IgG F(ab′) _2_ concentrations, with and without 600 nM α,β-meATP pretreatment.

The relative contribution of P2X1 receptors to collagen-evoked Ca ^2+^ responses compared with other pathways is greater at low compared with high levels of stimulation, ranging from approximately 45 to 92%, across a 10-fold concentration of agonist. 8 18 P2X1 receptors contributed significantly across the entire range of IgG F(ab′) _2_ concentrations tested ( Fig. 2 A–F ), with desensitization resulting in a reduction to 71% of control at 30 μg/mL; 67% of control at 15 μg/mL IgG F(ab′) _2_ ; 62% of control at 7.5 μg/mL IgG F(ab′) _2_ and 65% of control at 3.75 μg/mL IgG F(ab′) _2_ (using two-way ANOVA, followed by Bonferroni's multiple comparisons tests). Although the largest P2X1 receptor contribution was observed at the intermediate concentrations of IgG F(ab′) _2_ ( Fig. 2 C , D , F ), the response was very weak in some donors at or below 7.5 μg/mL. Therefore, 15 μg/mL was selected as a consistent stimulus for further assessment of the contribution of P2X1 receptors to FcγRIIa receptor signalling and function.

### FcγRIIa Receptor-Evoked [Ca ^2+^ ] _i_ Increases are Resistant to Elevated Apyrase Levels and Nitric Oxide, but not PGI _2_

**Fig. 3:**
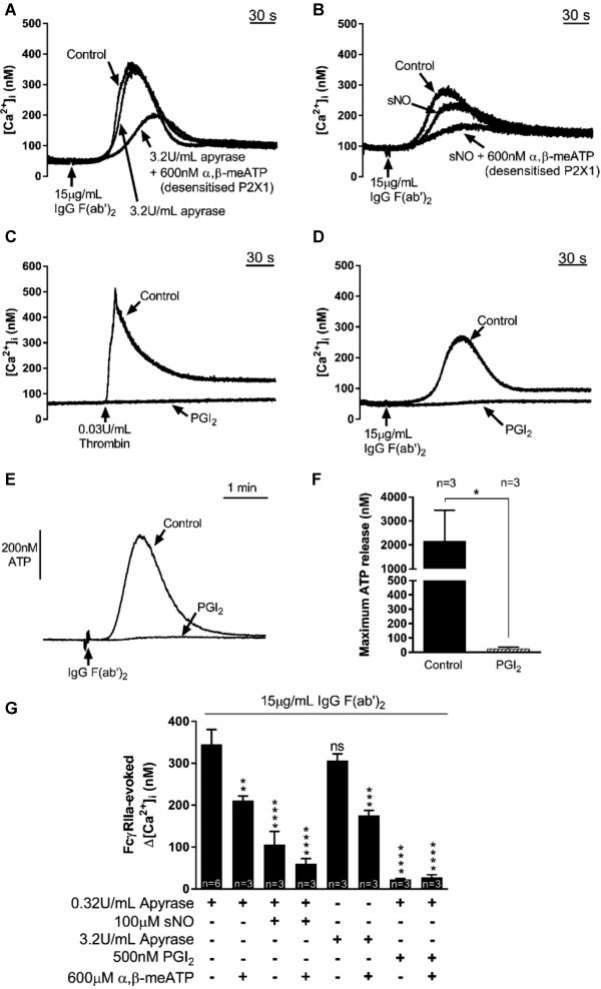
P2X1-mediated Ca ^2+^ responses to FcγRIIa receptor activation are resistant to NO and elevated apyrase levels, but are abolished by PGI _2_ . Representative [Ca ^2+^ ] _i_ responses ( **A, B, D** ) induced by FcγRIIa receptor activation by cross-linking of mAb IV.3 in the presence of elevated apyrase levels (3.2 U/mL), spermine NONOate (sNO; 100 μM), and prostacyclin (PGI _2_ ; 500 nM). Control traces are representative of the vehicle-treated platelet samples in each panel (saline, 0.01 M NaOH and dH _2_ O, respectively). P2X1 receptor desensitization was achieved by pretreatment with 600 nM α,β-meATP. ( **C** ) Effect of a submaximal concentration of PGI _2_ (500 nM) on [Ca ^2+^ ] _i_ induced by 0.03 U/mL thrombin. ( **E, F** ) PGI _2_ (500 nM) substantially inhibited FcγRIIa-mediated ATP secretion. ( **G** ) Average peak [Ca ^2+^ ] _i_ responses obtained in the presence of inhibitors used, compared with the average of control responses (first column).

In the intact circulation, platelets are constantly under the influence of inhibitory molecules, particularly endothelium-derived PGI _2_ and NO, and expression of the ectonucleotidase CD39 on the surface of endothelial cells. Elevating the levels of apyrase (from 0.32 to 3.2 U/mL) did not affect the FcγRIIa receptor-evoked Ca ^2+^ response and P2X1 receptors were still activated as a substantial reduction in the Ca ^2+^ response was observed, from 345 ± 35 nM to 175 ± 12 nM ( *p* < 0.05, *n* = 3, by one-way ANOVA, followed by Holm-Sidak's post hoc multiple comparisons), following pre-exposure to 600 nM α,β-meATP ( Fig. 3 A , G ). Endothelium-derived NO is known to regulate platelet activation mainly through elevation of cyclic GMP concentrations. 19 Pre-incubation of the platelet suspensions with a maximal concentration of the NO donor spermine NONOate (100 μM; unpublished data, Zeki Ilkan, 2017) caused a 69% reduction in the FcγRIIa receptor-induced Ca ^2+^ responses, from 345 ± 35 nM to 107 ± 31 nM (31% of control; *p* < 0.05, *n* = 3; Fig. 3 B ). The remaining response was virtually eliminated following pre-addition of α,β-meATP (to 60.0 ± 12.4 nM; *p* < 0.05, compared with control, *n* = 3, by one-way ANOVA, followed by Holm-Sidak's post hoc multiple comparisons), demonstrating the major contribution to the NO-resistant component by P2X1 receptors ( Fig. 3 B , G ). Previous studies have demonstrated that a maximal concentration of either NO or PGI _2_ is able to abolish [Ca ^2+^ ] _i_ increases evoked by thrombin and thromboxane A _2_ but only partially reduce responses to collagen and the TLR2/1 agonist Pam _3_ CSK _4_ . 10 In our studies, a concentration of PGI _2_ (500 nM) that abrogated the Ca ^2+^ response to 0.03 U/mL thrombin (Δ[Ca ^2+^ ] _i_ reduced from 431 ± 56 nM to 15.7 ± 6.2 nM; *p* < 0.05, *n* = 3, by paired *t* -test; Fig. 3 C ) also virtually eliminated the response via FcγRIIa receptors (345 ± 35 nM under control conditions to 23.0 ± 2.0 nM with PGI _2_ ; 7% of control; *p* < 0.05, *n* = 3, by one-way ANOVA, followed by Holm-Sidak's post hoc multiple comparisons; Fig. 3D, G ). This was unexpected since both GPVI and FcγRIIa signal through an ITAM-dependent pathway. The collagen-dependent stimulation of P2X1 receptors persists in high PGI _2_ due to a partial resistance of GPVI-evoked dense granule secretion to elevated cyclic AMP. 10 20 In contrast, FcγRIIa-induced ATP release was completely blocked by 500 nM PGI _2_ , thus accounting for its ability to prevent P2X1 receptor activation ( Fig. 3 E , F ).

### *Streptococcus sanguinis* Induces P2X1-Mediated [Ca ^2+^ ] _i_ Increases

**Fig. 4:**
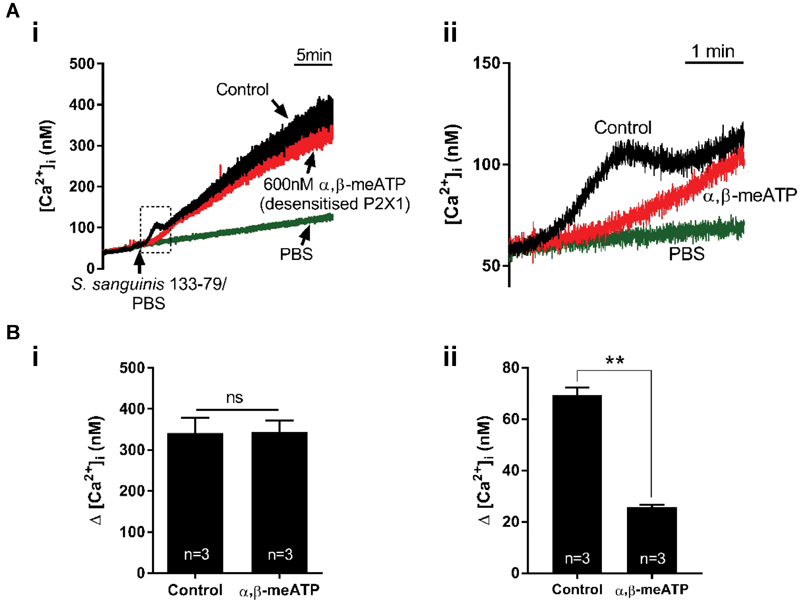
P2X1 ion channels contribute to the *S. sanguinis* -induced FcγRIIa receptor Ca ^2+^ responses ( **A** ) Representative Ca ^2+^ responses measured when *S. sanguinis* was added with and without 600 nM α,β-meATP pretreatment, together with the vehicle (PBS) control (i). α,β-meATP pretreatment diminished the transient Ca ^2+^ peak that occurred after bacterial stimulation [shown in dashed rectangular box in (i), which is expanded in (ii)]. ( **B** ) Average peak [Ca ^2+^ ] _i_ responses obtained within 25 minutes (i) and 2.5 minutes (ii) after bacterial stimulation, with and without α,β-meATP pretreatment.

A range of *Streptococci* strains including *S. sanguinis* have been previously shown to induce platelet aggregation in washed platelet suspensions in the presence of human IgGs. 5 Over a period of 25 minutes after addition of *S. sanguinis* , a steady increase in [Ca ^2+^ ] _i_ compared with vehicle control was observed, and the maximal Ca ^2+^ level reached at the end of the experiment was not significantly altered after desensitization of P2X1 channels using α,β-meATP (342 ± 37 nM for control and 344 ± 27 nM after α,β-meATP; *p* > 0.05, *n* = 3, using paired *t* -test; Fig. 4Ai, Bi ). However, a transient Ca ^2+^ increase was observed approximately 2.5 minutes after addition of bacteria, which was inhibited following P2X1 receptor desensitization (see expanded traces in Fig. 4 (Aii) corresponding to the dashed rectangular area indicated in Fig. 4Ai ). The peak [Ca ^2+^ ] _i_ increase during this initial transient was 69.2 ± 3.2 nM and reduced to 25.8 ± 0.9 nM ( *p* < 0.05, *n* = 3, using paired *t* -test) at the same time point in the paired P2X1 inhibited run, which represents a reduction to 37% of control ( Fig. 4Bii ). Thus, Ca ^2+^ entry through P2X1 channels is stimulated following FcγRIIa receptor activation either by bacteria or cross-linking antibodies.

### FcγRIIa Receptor Activation Induces Dense Granule Release and Aggregation that Partially Depends on P2X1 Responses

**Fig. 5:**
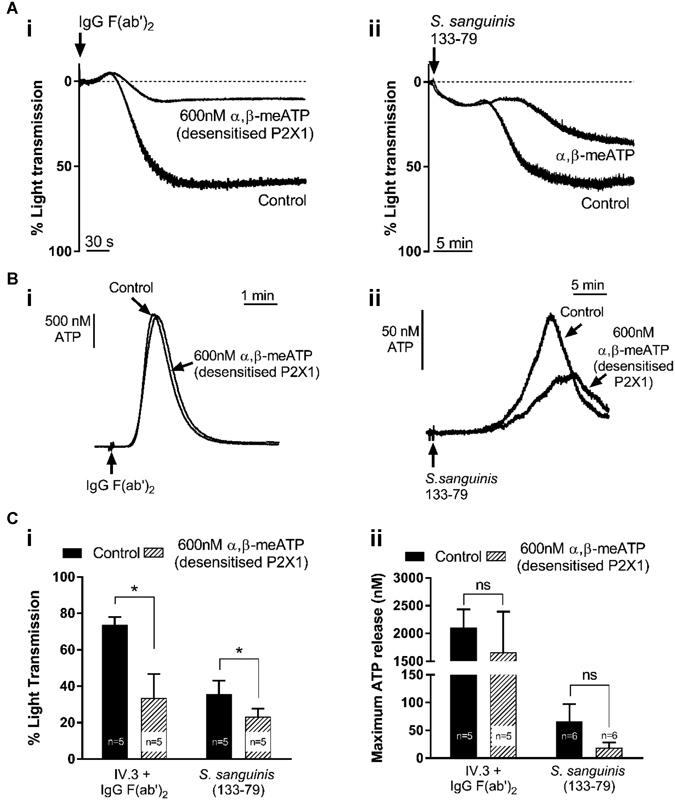
P2X1 channels contribute to aggregation induced by *S. sanguinis* and cross-linking of mAb IV.3 with IgG F(ab′) _2_ in washed platelets, and dense granule secretion is persistent to P2X1 desensitization. Representative aggregation traces ( **A** ), extracellular ATP elevations obtained using luciferin-luciferase assay ( **B** ), and average peak responses ( **Ci** and **Cii** ) obtained using *S. sanguinis* and cross-linking of mAb IV.3 to stimulate FcγRIIa receptor activation in the presence and absence of 600 nM α,β-meATP. In panels A and B, responses induced by cross-linking of mAb IV.3 are shown in (i) and by bacteria in (ii).

Platelet aggregation induced by either IgG F(ab′) _2_ antibody or *S. sanguinis* was significantly inhibited by α,β-meATP pre-treatment (from 73.8 ± 4.2% to 33.5 ± 13.2%: 45% of control; and from 35.7 ± 1.9% to 23.2 ± 4.4%: 65% of control, respectively; *p* < 0.05, *n* = 5 for both, by paired *t* -test; Fig. 5A, Ci ). Although the average peak ATP release was smaller after P2X1 desensitization ( Fig. 5B,Cii ), this was not statistically significant; the extracellular ATP increase following antibody-induced FcγRIIa activation was 2,108 ± 324 nM and 1,660 ± 732 nM, and following bacteria-induced FcγRIIa activation was 65.8 ± 31.5 nM and 18.6 ± 9.6 nM in the control and α,β-meATP pre-treated platelets, respectively ( *p* > 0.05, *n* = 5 and 6, using paired *t* -test). Thus, while dense granule secretion is known to be Ca ^2+^ -dependent and the early FcγRIIa-induced ATP release will stimulate Ca ^2+^ influx through P2X1 receptors, the amplification of aggregation by this cation channel does not involve a significant enhancement of dense granule secretion.

### Amplification of FcγRIIa Receptor Responses Is Independent of Initial Tyrosine Phosphorylation Events

**Fig. 6:**
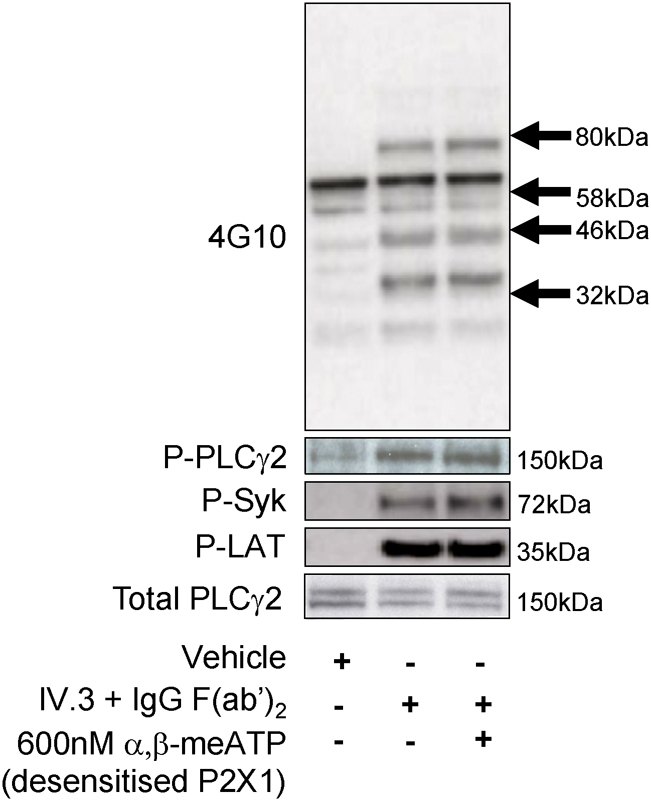
FcγRIIa receptor stimulation by cross-linking of mAb IV.3 induces phosphorylation events that are independent of P2X1 activation. Platelets were incubated for 60 seconds in the presence of vehicle (control), or IgG F(ab′) _2_ and mAb IV.3, following pretreatment with 600 nM α,β-meATP to desensitize P2X1 receptors. Samples were then lysed and analysed by Western blotting; note that the total sample was lysed; therefore, the total protein content will be consistent across all samples. This was shown by measurement of protein level (with 20 μg of total protein loaded per lane) and by re-probing for total PLCγ2. The pan-phosphotyrosine antibody 4G10 measures total tyrosine phosphorylation, while the following phospho-specific antibodies were used to measure phosphorylation of Syk, LAT and PLCγ2 at specific sites: phospho-Syk (Tyr 525/526), LAT (Tyr 200) and PLCγ2 (Tyr 1217). Results are representative of three experiments.

In addition to direct effects of Ca ^2+^ influx on functional events, we also considered the possibility that P2X1 receptors may enhance FcγRIIa receptor-induced responses through modulation of early tyrosine phosphorylation events. FcγRIIa-induced tyrosine phosphorylation was assessed across all sites using a pan-phosphotyrosine antibody (4G10) along with phosphorylation of specific targets using phospho-specific antibodies (Syk: Tyr 525/526; LAT: Tyr 200; PLCγ2: Tyr 1217). Samples were lysed 60 seconds after receptor stimulation, under control and P2X1-desensitizing conditions which represents the time point of maximal [Ca ^2+^ ] _i_ response to cross-linking with 15 μg/mL IgG F(ab′) _2_ . No observable difference in the attained phosphorylation levels after FcγRIIa receptor stimulation was observed following pretreatment with α,β-meATP ( Fig. 6 ).

## Discussion

ATP-gated P2X1 channels are the only ligand-gated Ca ^2+^ -permeable ion channels conclusively shown to be expressed on the platelet surface and provide a rapid route for Ca ^2+^ entry following ATP release from damaged vascular cells or from activated platelets and other blood cells. 7 21 22 These channels have been shown to increase the platelet [Ca ^2+^ ] _i_ responses observed in vitro following stimulation by several major haemostatic agonists, including ADP, collagen, thrombin and thromboxane A _2_ , and to exacerbate thrombosis in vivo. 8 9 10 11 In addition to their role in haemostasis, platelets are recognized to contribute to the immune system, assisting for example in the opsonization and thus clearance of bacteria. 23 24 25 26 27 Platelets have been shown to secrete cytokines, chemokines and antimicrobial substances such as β-defensin, which help facilitate leukocyte recruitment and assist in the elimination of invading pathogens through the formation of neutrophil extracellular traps (NETs). 26 27 28 The present study demonstrates that P2X1 ion channels also amplify the platelet [Ca ^2+^ ] _i_ and aggregation responses following stimulation of FcγRIIa, the only receptor in this cell type that recognizes the Fc region of IgG antibodies. Another immune receptor expressed on platelets is the TLR2/1 complex, which likewise uses ATP release and P2X1 receptors to enhance Ca ^2+^ responses and aggregation. 10 Therefore, by contributing to signalling downstream of both TLR2/1 receptors and FcγRIIa, P2X1 ion channels represent a route for Ca ^2+^ entry that can amplify platelet-dependent immune responses.

An increase in [Ca ^2+^ ] _i_ provides an essential signal that universally links surface receptors to inside-out activation of the main receptor for fibrinogen, GPIIb/IIIa, through activation of the Ca ^2+^ sensor CalDAG-GEFI. 29 30 31 Arman and colleagues have demonstrated that various IgG-coated bacterial strains, including *S. sanguinis* 133–79, induce GPIIb/IIIa-dependent tyrosine phosphorylation of FcγRIIa receptors. 5 P2X1 desensitization resulted in a more substantial reduction of FcγRIIa-evoked aggregation (by ∼55%, Fig. 5Ai , Ci ) than peak Ca ^2+^ increase (∼34%, Fig. 1C, E ), although this likely reflects the fact that [Ca ^2+^ ] _i_ activates functional events in a highly nonlinear manner and with a threshold above the resting concentration of approximately 100 nM. 32 However, the substantial proportion of the bacteria-induced aggregation that was dependent upon P2X1 receptors was surprising given the small size of the initial Ca ^2+^ increase induced by these cation channels following bacterial stimulation. It is possible that the timing of this early signalling event is crucial in the chain of events leading to inside-out activation of GPIIb/IIIa following bacteria-dependent engagement of FcγRIIa. A similar large inhibition of both Ca ^2+^ responses and aggregation is observed in human platelets at a low concentration of collagen, despite the small changes in [Ca ^2+^ ] _i_ . 9 12 18 An additional explanation could be that P2X1-dependent bacteria-induced inside-out activation of GPIIb/IIIa via FcγRIIa occurs within microdomains that display higher local [Ca ^2+^ ] _i_ increases. In support of this concept, single cell recordings from both platelets and megakaryocytes reveal that both ATP release and P2X1 receptor activation occur as a series of discrete transient events reflecting spatially restricted foci of dense granule content release and channel activation. 33 This spatial organization may result from the reported organization of P2X1 receptors, components of the secretory pathway and FcγRIIa-dependent signalling events into lipid rafts. 34 35 36

The efficiency with which P2X1 can amplify Ca ^2+^ and aggregation responses downstream of tyrosine kinase-coupled receptors such as FcγRIIa and GPVI receptors also raises the question of whether the cation channel enhances early kinase activation and/or granule secretion. However, no significant changes in FcγRIIa tyrosine phosphorylation events or bulk phase ATP secretion could be detected following inhibition of P2X1 receptors. It follows, therefore, that activation of P2X1 channels by FcγRIIa receptors and subsequent contribution to aggregation occur downstream of both PLCγ activation and initial release of ATP from dense granules. Maximal P2X1 receptor-dependent Ca ^2+^ influx is not able to independently cause aggregation, 37 thus must synergize with other cytosolic signals to enhance inside-out activation of GPIIb/IIIa. However, P2X1 receptors may enhance FcγRIIa-stimulated Ca ^2+^ mobilization via potentiation of IP _3_ receptors, as shown for P2Y1 receptors. 38 Pathways for Ca ^2+^ entry other than P2X1 also contribute to the FcγRIIa-induced [Ca ^2+^ ] _i_ increase ( Fig. 1 E ), which likely include Orai1 store-operated Ca ^2+^ channels 39 40 and/or TRPC6 stimulated by either diacylglycerol or a decrease in PIP _2_ . 41 Nevertheless, the autocrine activation of P2X1 by secreted ATP permits a very efficient early mechanism for Ca ^2+^ influx that contributes distinctly and separately from other Ca ^2+^ entry pathways. 10

Platelet FcγRIIa represents the largest pool of these receptors in the body due to the large number of circulating platelets compared with other immune-competent cells expressing such receptors. 1 42 43 FcγRIIa can directly activate platelets following the binding of IgG-containing complexes, certain strains of IgG-coated bacteria and unidentified ligand(s) on cancer cells. 5 26 Innate pentraxins such as C-reactive protein can also activate FcγRIIa receptors, at least in leukocytes. 44 FcγRIIa receptors play a particularly important role in heparin-induced thrombocytopenia (HIT). 45 46 Despite the reduced platelet count, thrombosis occurs in HIT due to direct activation of FcγRIIa receptors on platelets in combination with monocyte-dependent generation of thrombin and tissue factor. 45 It has also been shown that FcγRIIa receptors amplify platelet activation by weak levels of haemostatic agonists such as thrombin and thromboxane A _2_ , which may contribute to the prothrombotic phenotype in HIT. 47 It is therefore interesting to speculate that P2X1 receptor inhibition could serve as a useful antithrombotic therapy during immune thrombocytopenia, particularly since P2X1 ^−/−^ mice display no significant bleeding phenotype. 9

An important property of the [Ca ^2+^ ] _i_ increases evoked by both GPVI and TLR2/1 is their partial resistance to inhibition by NO and PGI _2_ even at low levels of stimulation, since this permits activation even in the intact circulation. 10 20 This contrasts with an ability of both these cyclic nucleotide-elevating endogenous platelet inhibitors to totally abolish Ca ^2+^ responses mediated by several G-protein–coupled receptors. This can be explained by a difference in the cyclic nucleotide sensitivity of secretion downstream of GPCRs compared with tyrosine kinase-linked receptors such as GPVI and TLR2/1. 10 20 P2X1 receptors remain unaffected by cyclic nucleotide elevation and continue to be activated by the remaining amounts of ATP released. Like GPVI, FcγRIIa receptors signal through an immunoreceptor-based tyrosine ITAM motif and activation of the Src/Syk/LAT/PLCγ2 signalling cascade. 2 48 Thus, it is not surprising that Ca ^2+^ responses downstream of FcγRIIa receptors were also partially resistant to NO in part due to continued P2X1 receptor involvement ( Fig. 3B, G ). However, in contrast, PGI _2_ completely abolished FcγRIIa-evoked secretion and Ca ^2+^ responses ( Fig. 3D–G ). Furthermore, this was not due to a weaker secretory response for FcγRIIa, since antibody-induced ATP release was substantially larger than observed previously for GPVI under the same conditions ( Fig. 5Bi ). 10 The large ATP release in combination with efficient autocrine P2X1 activation can explain why FcγRIIa-evoked Ca ^2+^ increases were insensitive to elevated apyrase compared with a significant ability of this ectonucleotidase to reduce P2X1-stimulated Ca ^2+^ increases after GPVI engagement. 10 At present, we cannot explain the difference in PGI _2_ sensitivity of GPVI versus FcγRIIa secretion and Ca ^2+^ mobilization other than to speculate that the downstream signalling events are sufficiently distinct in their susceptibility to cyclic AMP or protein kinase A. This may result from the differences in coupling of the external ligand to ITAM domain activation, since the FcγRIIa receptor contains the tyrosine motif within its intracellular tail, whereas GPVI needs to associate with FcRγ to achieve ITAM-induced signalling. The FcγRIIa and FcRγ ITAM domains also differ in structure. A further site for differential cyclic AMP/PKA-dependent inhibition could be at the level of exocytosis. It is becoming clear that different modes of exocytosis exist in platelets, such as single and multigranular fusion events, which could allow specificity amongst individual agonists. 49 50

In conclusion, the present study provides evidence that ATP-gated P2X1 channels amplify the intracellular Ca ^2+^ and aggregation responses in human platelets following stimulation of FcγRIIa receptors. FcγRIIa-induced P2X1 activation was resistant to increased ectonucleotidase activity and persisted following an increase in the presence of the endogenous platelet inhibitor NO, which may allow immune complexes or opsonized bacteria to stimulate platelets in the intact circulation. This raises the possibility that inhibition of P2X1 receptors could represent an antithrombotic target during immune thrombocytopaenia or inflammatory situations such as IE.

## Notes

What is known about this topic?

- Platelets express FcγRIIa receptors which signal through tyrosine kinases.
- An increase in intracellular Ca ^2+^ is an essential signalling event during platelet activation; however, the relative importance of different Ca ^2+^ entry pathways to FcγRIIa receptor signalling is unknown.

**What does this paper add?**

- Stimulation of FcγRIIa receptors by antibodies or bacteria leads to Ca ^2+^ mobilization and aggregation, both of which are amplified by activation of P2X1 receptors following ATP release.
- P2X1 receptors enhance FcγRIIa-evoked responses downstream of tyrosine phosphorylation.
- FcγRIIa-dependent P2X1 receptor platelet signals may enhance innate immune responses in vivo.
